# Rare hamartomatous lesion in the inframammary region

**DOI:** 10.1016/j.jdcr.2024.12.033

**Published:** 2025-01-24

**Authors:** Clarisse Kaori Fujishige, Letícia Turolla Leal, Isabella Zurita Dehó, Gabrielle Ellert de Almeida, Lucas Giordani, Beatrice Martinez Zugaib Abdalla, Gilles Landman, Francisco Macedo Paschoal, Eduardo Lacaz Martins

**Affiliations:** a University Center of the ABC School of Medicine - Dermatology, São Paulo, Brazil; b Hospital Sírio-Libanês - Dermatology, São Paulo, Brazil; c Gilles Laboratory, São Paulo, Brazil; d Federal University of São Paulo - Dermatology, São Paulo, Brazil

**Keywords:** benign, connective tissue nevus of the proteoglycan type, hamartoma, mucinous nevus

## Case report

**Fig 1:**
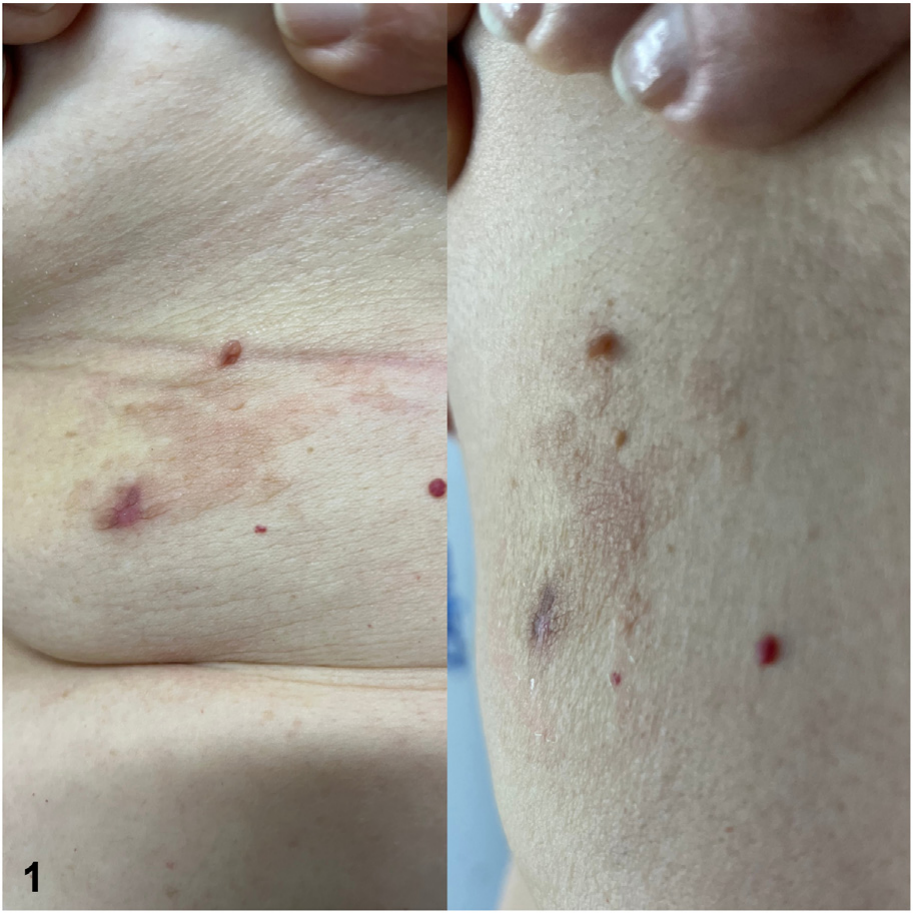

A 66-year-old woman patient, phototype III, born in São Paulo, Brazil, complained of a lesion that appeared 2 years prior, on the right inframammary region. On examination, there were discreetly erythematous-brown confluent papules forming a plaque with a rough surface, measuring 4 × 8 cm (Fig 1). The personal medical history was positive for osteoporosis, hypothyroidism, and osteoarthritis (currently using levothyroxine at an appropriate dose according to laboratory control).

**Fig 2:**
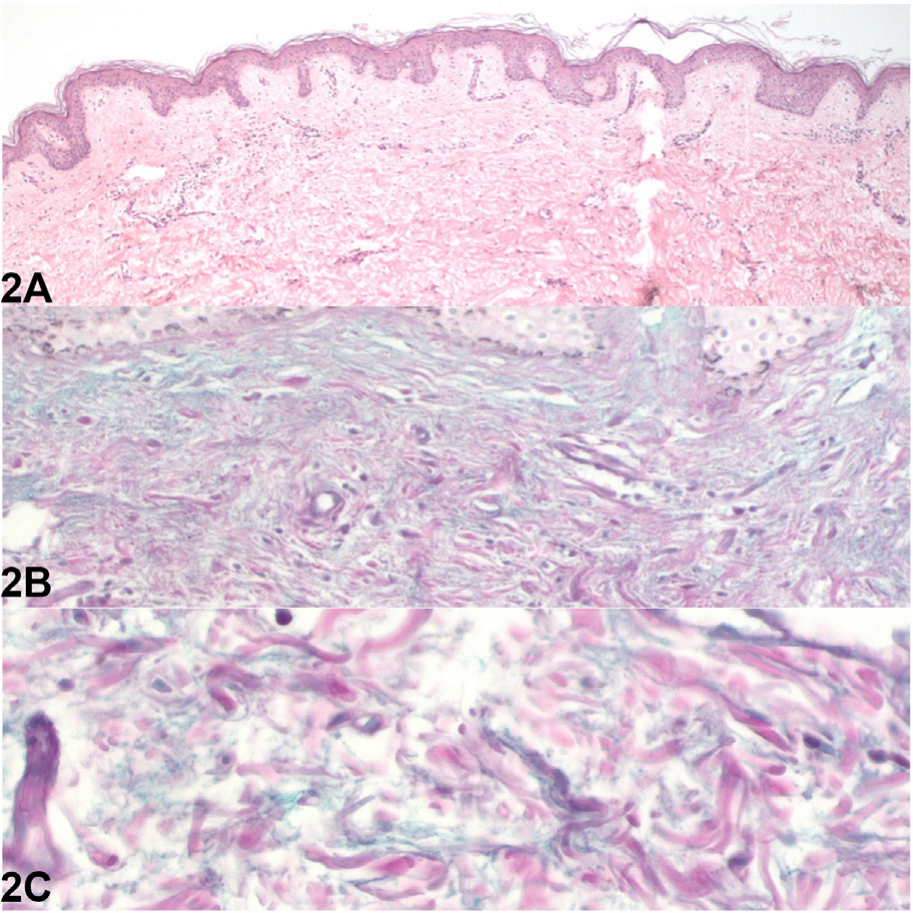

**Question 1: What is the diagnosis?**

A. Cutaneous myxoma
B. Cutaneous pilar leiomyoma
C. Mucinous nevus
D. Nodular amyloidosis
E. Shagreen patch

**Answers:**

A. Cutaneous myxoma – Incorrect. Cutaneous myxomas usually present as nodules and may be accompanied by other findings of the Carney complex.^1^
B. Cutaneous pilar leiomyoma – Incorrect. These are usually painful and tender.^1^
C. Mucinous nevus – Correct. Skin-colored or slightly pigmented papules that group into plaques, with unilateral and dermatomal distribution.^1^
D. Nodular amyloidosis – Incorrect. Rare; it usually appears as nodules or plaques with a brownish-erythematous color.^1^
E. Shagreen patch – Incorrect. A typical collagenoma of tuberous sclerosis; the patient in question presents an isolated lesion without other findings.^1^

**Question 2: What histopathologic findings characterize the lesion?**

A. Hyperkeratosis and acanthosis with elongation of the epidermal papillae and mucin deposition in the papillary and mid dermis
B. Lymphocytic inflammatory infiltrate
C. Normal epidermis with mucin deposition in the papillary and mid dermis
D. Some histologic findings can only be seen with Alcian blue at pH 2.5 and colloidal iron
E. All alternatives are correct

**Answers:**

A. Hyperkeratosis and acanthosis with elongation of the epidermal papillae and mucin deposition in the papillary and mid dermis – Correct. This is the description of the mucinous nevus, specifically the subtype of combined mucinous nevus (combined epidermal—connective tissue nevus of the proteoglycan [CTNP] type).^2^
B. Lymphocytic inflammatory infiltrate – Correct. A mild perivascular lymphocytic inflammatory infiltrate with mild edema can be found in a mucinous nevus (Fig 2, *B*).^2^
C. Normal epidermis with mucin deposition in the papillary and mid dermis – Correct. This is the description of the mucinous nevus, specifically the subtype of pure dermal mucinous nevus (CTNP), the subtype presented by the patient described in the clinical case according to the anatomopathological images (Fig 2, *A*).^2^
D. Some histologic findings can only be seen with Alcian blue at pH 2.5 and colloidal iron – Correct. Both stains allow visualization of the mucin (Fig 2, *C*). Additionally, they are sensitive to hyaluronidase and negative for periodic acid–schiff.^3^
E. All alternatives are correct – Correct. All the statements describe a mucinous nevus; it is important to differentiate the 2 subtypes: connective tissue nevus of the proteoglycan—CTNP and combined epidermal—CTNP type.^4^

**Question 3: Which is the most indicated treatment for mucinous nevus according to the literature?**

A. Electrodesiccation
B. Follow-up considering the benign nature of the lesion
C. Surgical excision
D. Topical corticosteroids and retinoids
E. Vaporization with a CO_2_ laser

**Answers:**

A. Electrodesiccation – Incorrect. No reports of recurrences, but with risk of local scarring.
B. Follow-up considering the benign nature of the lesion – Correct. As it is a benign and asymptomatic disease, no treatment is necessary, being the most indicated treatment, except for aesthetic purposes.^5^
C. Surgical excision – Incorrect. No reports of recurrences, but with risk of local scarring.
D. Topical corticosteroids and retinoids – Incorrect. Corticosteroids and retinoids have been described as having no effect.^2^
E. Vaporization with a CO_2_ laser – Incorrect. In cases of pure dermal nevus, caution is advised, as the epidermis is normal and can produce scars.

## Conflicts of interest

None disclosed.
